# Rice Bran Protein Hydrolysates Improve Insulin Resistance and Decrease Pro-inflammatory Cytokine Gene Expression in Rats Fed a High Carbohydrate-High Fat Diet

**DOI:** 10.3390/nu7085292

**Published:** 2015-08-03

**Authors:** Kampeebhorn Boonloh, Veerapol Kukongviriyapan, Bunkerd Kongyingyoes, Upa Kukongviriyapan, Supawan Thawornchinsombut, Patchareewan Pannangpetch

**Affiliations:** 1 Department of Pharmacology, Faculty of Medicine, Khon Kaen University, 123 Mittraparp Highway, Muang District, Khon Kaen 40002, Thailand; E-Mails: kampeeporn@yahoo.com (K.B.); veerapol@kku.ac.th (V.K.); bunkon@kku.ac.th (B.K.); 2 Department of Physiology, Faculty of Medicine, Khon Kaen University, Khon Kaen 40002, Thailand; E-Mail: upa_ku@kku.ac.th; 3 Department of Food technology, Faculty of Technology, Khon Kaen University, Khon Kaen 40002, Thailand; E-Mail: suptha@kku.ac.th

**Keywords:** metabolic syndrome, insulin resistance, rice bran protein hydrolysates (RBP), inflammatory cytokines

## Abstract

A high carbohydrate-high fat (HCHF) diet causes insulin resistance (IR) and metabolic syndrome (MS). Rice bran has been demonstrated to have anti-dyslipidemic and anti-atherogenic properties in an obese mouse model. In the present study, we investigated the beneficial effects of rice bran protein hydrolysates (RBP) in HCHF-induced MS rats. After 12 weeks on this diet, the HCHF-fed group was divided into four subgroups, which were orally administered RBP 100 or 500 mg/kg, pioglitazone 10 mg/kg, or tap water for a further 6 weeks. Compared with normal diet control group, the MS rats had elevated levels of blood glucose, lipid, insulin, and HOMA-IR. Treatment with RBP significantly alleviated all those changes and restored insulin sensitivity. Additionally, RBP treatment increased adiponectin and suppressed leptin levels. Expression of *Ppar-*γ mRNA in adipose tissues was significantly increased whereas expression of lipogenic genes *Srebf1* and *Fasn* was significantly decreased. Levels of mRNA of proinflammatory cytokines, *Il-6*, *Tnf-*α, *Nos-2* and *Mcp-1* were significantly decreased. In conclusion, the present findings support the consumption of RBP as a functional food to improve insulin resistance and to prevent the development of metabolic syndrome.

## 1. Introduction

Excessive consumption of fat, fructose or carbohydrates leads to disturbances in fatty acid and carbohydrate metabolism [1,2]. This is accompanied by an increase in fat deposits and subsequent development of a proinflammatory state in adipose tissue and reduced fatty acid oxidation [3]. In particular, the accumulation of visceral fat is commonly linked to insulin resistance (IR), metabolic syndrome (MS), atherosclerosis and type 2 diabetes mellitus (T2DM) [4,5,6].

Diets high in carbohydrates and fats cause an increased influx of free fatty acids (FFA), which also promote intra-hepatic and intra-myocellular triglyceride accumulation resulting in impairment of insulin signaling in non-adipose tissue [7,8]. FFAs and lipid-derived mediators are ligands for immune receptors such as Toll-like receptors and G-protein coupled receptors and can thus initiate an innate immune response [9] to induce the increase of inflammatory cytokines such as tumor necrosis factor-α (TNF-α), interleukin-6 (IL-6), monocyte chemoattractant protein-1 (MCP-1), and plasminogen activator inhibitor-1 (PAI-1) as seen in obesity and IR [10,11]. These proinflammatory cytokines exert a paracrine effect to downregulate insulin signaling [9,10,11] via STAT3, SOCS3 and JNK signaling systems [12]. In addition, TNF-α has been shown to downregulate the expressions of adiponectin, insulin receptor substrate-1 (IRS-1) and peroxisome proliferator-activated receptor γ (Ppar-γ) in adipocytes [13], resulting in elevation of insulin and leptin levels but a decrease of adiponectin [14,15].

Adiponectin is secreted entirely by adipocytes and its level is inversely correlated with IR in lipodystrophy and obesity, and with inflammatory states [16]. Adiponectin increases glucose uptake and fatty acid oxidation, and inhibits hepatic gluconeogenesis [10,17]. Additionally, adiponectin acts as an anti-inflammatory by suppressing secretion of TNF-α [17] through inhibition of NF-κB [18]. The chronic overloading of adipocytes induces a chronic inflammatory state in the adipose tissue, manifested by recruitment of macrophages and other inflammatory cells into the adipose tissue to feed-forward the release of adipokines and inflammatory cytokines such as Mcp-1, TNF-α, IL-1β and IL-6 [19]. Furthermore, the chronic stress can upregulate sterol regulatory element-binding proteins-1c (SREBP-1c) [20]. SREBP-1c, which is encoded by the *Srebf1* gene, is the most important transcription factor regulating genes involved in *de novo* lipogenesis [21]. The regulation of SREBP-1c is also dependent on nutritional status and many other factors. It has been reported that long-term feeding with fructose or high fat can activate expression of *Srebf1* in the liver of rats [22], leading to rapid stimulation of lipogenesis with accumulation of triglyceride (TG), and contributes to hepatic IR [23].

Rice bran, a byproduct from the rice milling process derived from the outer layer of the rice grain, contains a number of nutrients and biologically active compounds. Interestingly, antidiabetic and antidyslipidemic activities of rice bran have been reported both in *in vivo* and *in vitro* experiments [24,25,26]. The lipid fraction of rice bran, particularly oryzanol has demonstrated a variety of biological effects, including cholesterol-lowering, anti-inflammatory, anti-cancer, anti-oxidant and anti-diabetic activities [27]. The protein content in Hom Mali rice bran is about 10%–15% from our recent report [24]. Rice bran protein has been found to be of high quality and to have significant food and pharmaceutical applications [28]. However, still there is limited information on the effect of the protein in rice bran on the regulation of glucose and lipid blood levels in metabolic syndrome. Therefore, in this study we investigated the effects of rice bran protein hydrolysates (RBP) on glucose and lipid dysregulation, insulin resistance, adipokine secretions and inflammatory profiles in rats with metabolic syndrome condition. We found that RBP could improve glucose and lipid homeostasis and insulin resistance, with increased adiponectin secretion and suppressed inflammatory cytokine secretion.

## 2. Materials and Methods

### 2.1. Chemical Reagents

Pioglitazone was purchased from Takeda Pharmaceutical (Osaka, Japan). Rat/mouse insulin, adiponectin and leptin ELISA Kits were obtained from Millipore (Life Sciences/Biotech, Petaluma, MA, USA). Trizol^®^ reagent was obtained from Invitrogen (Life Techologies, Eugene, OR, USA). iScript Reverse Transcription Supermix and Ssofast EvaGreen Supermix were obtained from BIO-RAD (Hercules, CA, USA). The hand-held glucometer (ACCU-CHEK^®^) was purchased from Roche diagnostics (Mannheim, Germany).

### 2.2. Rice Bran Protein Hydrolysates (RBP) Preparation

Cold-pressed defatted rice bran was obtained from The Organic Agriculture Community Enterprise, Lopburi province, Thailand. Rice bran protein hydrolysates were prepared according to a report by Thawornchinsombut and Kokkaew [29]. Briefly, protein was extracted from defatted rice bran by alkaline solubilization (pH 11.0) followed by pI precipitation (pH 4.5). Proteolysis using a commercial enzyme, Protease G6 (Genencor International Inc.^®^, Rochester, NY, USA) was performed at 3% E/S at pH 8.0, 55 °C for 4 h. The enzyme was then inactivated at 85 °C for 15 min. After centrifugation, the protein hydrolysates were freeze-dried to obtain RBP powder. A yield of 8.8% RBP powder (based on weight of defatted rice bran) was achieved.

### 2.3. Animals and Experimental Protocols

Male Sprague-Dawley rats weighing 220–230 grams were obtained from the National Laboratory Animal Center, Mahidol University, Thailand. All animal experimental protocols were approved by the Animal Ethics Committee of Khon Kaen University (AEKKU 40/2555). After seven days of acclimatization, animals were randomly assigned into five groups with seven rats in each group. The first group, the normal control group, received a normal chow diet and sterile tap water (SW) ad libitum throughout the experimental period. An insulin-resistant condition was induced in the remaining rats by feeding them a HCHF diet according to the study by Panchal et al [30] with some modification (the composition of HCHF is shown in Table 1). Fructose (15% in drinking water) was given for the whole period of the experiment to the HCHF-fed rats. After 12 weeks, the animals in the HCHF-diet groups were daily given orally SW (HCHF-control group), RBP 100 or 500 mg/kg, or pioglitazone 10 mg/kg, an insulin sensitizing agent (for the positive control group) for a further 6 weeks. The RBP and pioglitazone solutions were freshly prepared and administered orally using an oro-gastric feeding needle. To determine the oral dosage, the animals were weighed before each administration.

**Table 1:** Composition of normal- and high-carbohydrate and high-fat diets.

Compositions	Normal Chow (g/Kg)	HCHF Diet (g/Kg)
Powdered rat food *	945	200
Fructose	-	400
Lard oil	-	200
Sweetened condensed milk	-	145
Mineral salts and vitamins	25	25
Fiber	30	30
Energy (Kcal/Kg)	3040	4230

* The powdered rat food (CP Mouse Feed, Bangkok, Thailand) composed of 24% protein, 4.5% fat, 56% carbohydrate, 5% fiber and moisture.

### 2.4. Determinations of Fasting Blood Glucose, Glucose Tolerance and Insulin Resistance

At day 4 of the sixth week of treatments, fasting blood glucose (FBG), glucose tolerance test (OGTT) and insulin resistance were determined in fasting rats. The animals were deprived of food for 14 h but had free access to water. Blood was collected from the tail vein for glucose analysis before glucose loading (FBG at 0 min) and 30, 60 and 120 min after oral administration of glucose solution (2 g/kg). The area under the curve (AUC) of time *vs.* blood glucose concentrations, which indicated the total amount of blood glucose from 0 to 120 min, was determined. The AUC is calculated by the trapezoidal method.

Serum insulin level of fasting blood was measured and insulin resistance was evaluated according to the homeostasis model assessment (HOMA) method described by Matthews *et al.* [31]. The HOMA-IR index was calculated as follows: (fasting insulin (μIU/mL) × fasting glycemia (μmol/L))/22.5. Blood glucose was examined using a glucometer (ACCU-CHEK^®^). Insulin level was measured using a rat/mouse ELISA kit (Millipore^®^, Life Sciences/Biotech, Petaluma, MA, USA).

### 2.5. Determinations of Lipid Profiles, Adiponectin and Leptin

At the end of all treatments, fasted animals were anesthetized by sodium pentobarbitone (60 mg/kg, intraperitoneal injection). Blood samples were collected from the abdominal aorta for determination of lipid profile (TC, TG, LDL and HDL), adiponectin and leptin. Serum adiponectin and leptin were measured using ELISA kits (Millipore, Life Sciences/Biotech, Petaluma, MA, USA). Lipid profiles were measured using enzymatic and colorimetric methods (Roche diagnostics, Bangkok, Thailand).

### 2.6. Analysis of mRNA Expression by Real-Time Quantitative Reverse Transcription Polymerase Chain Reaction (RT-qPCR)

At the end of all treatments, the liver of each rat was immediately removed and stored at −80 °C for subsequent RNA extraction for determination of expression levels of Srebf1 and Fasn. Similarly, visceral fat tissue was collected for determination of Ppar-γ and the proinflammatory cytokine genes, Il-6, Tnf-α, Nos2 and Mcp-1, and the anti-inflammmatory cytokine gene, Il-10.

Total RNA was extracted from frozen livers and white adipose tissues using TRIzol^®^ reagents according to the manufacturer’s instructions. First-strand complementary DNA (cDNA) was synthesized from 1 µg of total RNA using iScript reverse transcriptase at 25 °C for 5 min, 42 °C for 30 min, with a final step of 5 min at 85 °C in a C1000 Thermal cycler (Bio-RAD, Hercules, CA, USA). The RT-qPCR analysis was performed as described previously [32] using cDNA template, 0.5 μM of each primer and SsoFast EvaGreen Supermix (7.5 µL) in a final reaction volume of 15 µL. PCR reactions were performed in a LightCycler^®^480 Real-Time PCR instrument (Roche Applied Science). Samples were incubated in the light cycler apparatus for an initial denaturation step at 95 °C for 3 min, followed by 40 cycles of denaturation step at 95 °C for 15 s and extension step at 72 °C for 30 s. The specific primers used are described in Table 2. Levels of specific mRNAs were expressed relative to β*-actin*. Relative fold change for target mRNA was calculated using the standard curve method. Amplification of specific transcripts was confirmed by melting curves profiles generated at the end of each run.

**Table 2:** Nucleotide sequences of primers used for PCR *(Rattus norvegicus).*

Genes	Forward Primer	Reverse Primer	PCR Product
**β-actin** (NM_031144.3)	5′-GGAGATTACTGCCCTGGCTCCTA-3′	5′-GACTCATCGTACTCCTGCTTGCTG-3′	150 bp
***Il-6*** (NM_012589.2)	5′-GAAGTTTCTCTCCGCAAGAGACTT-3′	5′-ACATATGTAATTAAGCCTCCGACTTGT-3′	171 bp
***Il-10*** (NM_012854.2)	5′-CCTCTGGATACAGCTGCGA-3′	5′-TGTCACGTAGGCTTCTATGC-3′	166 bp
***Tnf-*α** (NM_012675.3)	5′-GTAGCCCACGTCGTAGCAAAC-3′	5′-ACCACCAGTTGGTTGTCTTTGA-3′	113 bp
***Mcp-1*** (NM_031530.1)	5'-TGTCTCAGCCAGATGCAGTTAAT-3'	5'-CCGACTCATTGGGATCATCTT-3'	77 bp
***Nos2*** (NM_012611.3)	5′-AACCCAAGGTCTACGTTCAAG-3′	5′-AAAGTGGTAGCCACATCCCG-3′	133 bp
***Srebf1*** (NM_001276707.1)	5′-CCGAGGTGTGCGAAATGG-3′	5′-TTGATGAGCTGAAGCATGTCTTC-3′	64 bp
***Fasn*** (NM_017332.1)	5′-TCGACCTGCTGACGTCTATG-3′	5′-TCTTCCCAGGACAAACCAAC-3′	196 bp
***Ppar-*γ** (NM_013124.3) (NM_001145366.1) (NM_001145367.1)	5'- ATTCTGGCCCACCAACTTCGG-3'	5'- TGGAAGCCTGATGCTTTATCCCCA-3'	339 bp

### 2.7. Statistical Analysis

All values are expressed as means ± standard error of the mean (SE). Differences between mean values of normally distributed data were assessed with one-way analysis of variance (ANOVA) and Tukey’s post-hoc test. A *p*-value <0.05 was considered significant. The statistical analyses were performed using computer-based software Stata version 10 (College Station, TX, USA).

## 3. Results

### 3.1. Effect of RBP on Food Intake, Body Weight and Organ Weight

The normal control rats had a higher food intake than HCHF-fed rats, so consequently had higher body weights (25%–30%) throughout the experimental period. After feeding with HCHF diet for twelve weeks, rats were divided into four groups for applying various treatments for a further six weeks. The average initial body weight (at week 12) of each group of HCHF-fed rats was similar and was in the range of 316–332 g. After six weeks of treatments, the body weight of HCHF-rats treated with pioglitazone was significantly higher than that of the HCHF-control and HCHF-RBP rats.

Although HCHF-fed rats had less body weight than normal controls, the relative weights of their livers were significantly greater, as were those of retroperitoneal fat tissues (an indicator of metabolic syndrome) (Table 3). The daily oral administration of RBP (500 mg/kg) or pioglitazone (10 mg/kg) caused a significant decrease in relative weights of livers but not of fat (Table 3).

**Table 3:** The effect of RBP on body weights and relative weights of livers and white adipose tissues in HCHF-fed rats.

Groups	Liver (g/Kg BW)	Retroperitoneal Fat (White Adipose Tissue) (g/Kg BW)	Body Weight (g)
**Normal control**	31.9 ± 0.7	16.7 ± 0.7	512.8 ± 4.3
**HCHF control**	39.8 ± 0.1 *	21.9 ± 1.1 *	370.0 ± 4.5 **
**HCHF-RBP100**	37.6 ± 0.7 *	18.7 ± 0.6	367.8 ± 4.1 **
**HCHF-RBP500**	33.4 ± 0.2 ^#^	18.5 ± 0.2	362.7 ± 3.8 **
**HCHF-Pioglitazone**	31.2 ± 0.9 ^#^	20.9 ± 0.8 *	405.1 ± 4.7 **

(All values are expressed as means ± SE. *: *p* < 0.05, significant increase as compared to normal controls; **: *p* < 0.05, significant decrease as compared to normal controls; ^#^: *p* < 0.05, significant decrease as compared to HCHF-control group, RBP: Rice bran protein hydrolysates, *n* = 7/group).

### 3.2. Effect of RBP on FBG, OGTT and Serum Lipid Profiles

HCHF-fed rats had elevated FBG and impaired OGTT, as indicated by an increased AUC (Table 4), which are among the characteristics of metabolic syndrome. In the group treated with RBP 500 mg/kg, FBG and AUC were significantly lower than in the HCHF-control group (Table 4) and similar to the group treated with pioglitazone 10 mg/kg.

**Figure 1:**
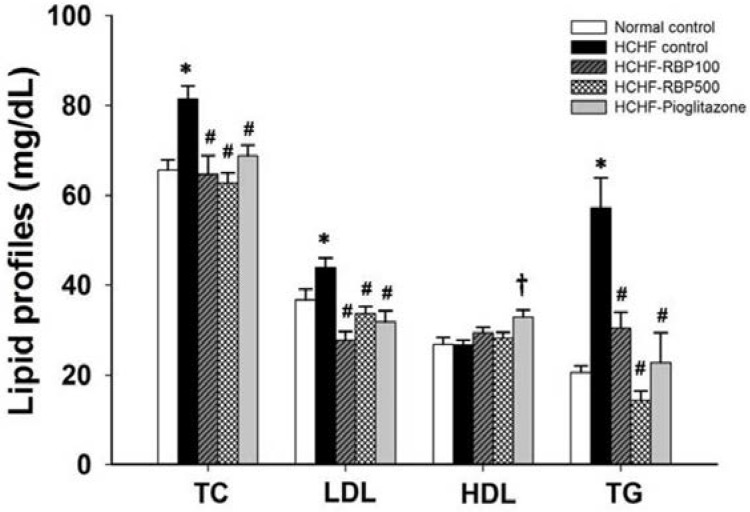
Effect of RBP on serum lipid profiles in HCHF-fed rats. Oral administration of RBP 100 or 500 mg/kg or pioglitazone 10 mg/kg daily for 6 weeks significantly decreased TC, LDL and TG. (*: *p* < 0.05, significant increase as compared to normal controls; ^#^: *p* < 0.05, significant decrease as compared to HCHF-control group; ^†^: *p* < 0.05, significant increase as compared to HCHF-control group, *n* = 7/group).

The HCHF-control group also showed markedly higher levels of serum TC, LDL-C and TG as compared with the normal control group, approximately by 25%, 20% and 178%, respectively (Figure 1). The RBP (100 and 500 mg/kg) and pioglitazone 10 mg/kg treatments significantly decreased TC, LDL-C and TG of HCHF-fed rats. RBP at 500 mg/kg was more effective at decreasing blood triglyceride levels than was pioglitazone. However, pioglitazone, but not RBP treatment, caused an increase in HDL-C (Figure 1).

**Table 4:** Effect of RBP on FBG and OGTT in HCHF- rats.

Groups	Fasting Blood Glucose (mg/dL)		AUC of OGTT (mg.min/dL)	
	Before Treatment	After Treatment	Before Treatment	After Treatment
**Normal control**	89.6 ± 7.2	92.2 ± 6.4	15,608.6 ± 607.7	16,114.3 ± 517.8
**HCHF control**	115.0 ± 9.1 *	119.5 ± 6.6 *	17,380.7 ± 841.5 *	17,959.8 ± 696.0 *
**HCHF-RBP100**	113.2 ± 7.3 *	116.4 ± 6.0	17,779.3 ± 803.6 *	17,628.6 ± 827.9 *
**HCHF-RBP500**	109.2 ± 10.6 *	99.7 ± 12.4 **^#^**	17,121.4 ± 1271.9 *	16,317.9 ± 596.3 **^#^**
**HCHF-Pioglitazone**	107.8 ± 12.2 *	75.2 ± 8.7 **^#^**	17,547.9 ± 910.4 *	15,910.7 ± 776.9 **^#^**

(All values are expressed as means ± SE. *: *p* < 0.05, significant increase as compared to normal controls; ^#^: *p* < 0.05, significant decrease as compared to HCHF-control group, RBP: Rice bran protein hydrolysates, *n* = 7/group).

### 3.3. Effect of RBP on Insulin Secretion and HOMA-IR

**Figure 2:**
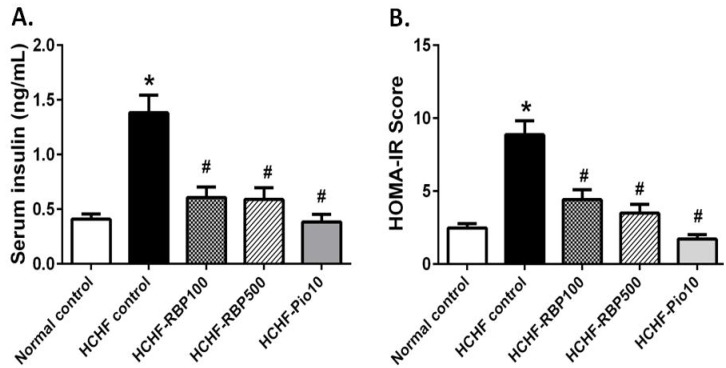
Effects of RBP on serum insulin levels (**A**) and HOMAR-IR values (**B**) in HCHF-fed rats. Oral administration of RBP 100 or 500 mg/kg or pioglitazone 10 mg/kg daily for 6 weeks significantly decreased serum insulin and HOMA-IR. (*: *p* < 0.05, significant increase as compared to normal controls; ^#^: *p* < 0.05, significant decrease as compared to HCHF-control group, *n* = 7/group).

Insulin resistance is an important characteristic of metabolic syndrome. In our study, animals fed the HCHF diet exhibited increases in serum insulin and HOMA-IR (Figure 2A,B). RBP 100 and 500 mg/kg or pioglitazone significantly decreased the elevated levels of insulin and especially the HOMA-IR to values similar to those in normal control rats (Figure 2A,B).

### 3.4. Effect of RBP on Adiponectin and Leptin Secretions

To investigate the effect of RBP on the regulatory mechanism of hyperglycemia and insulin resistance in more detail, we examined the levels of the two major blood glucose and lipid regulating adipokines, adiponectin and leptin, in the plasma of HCHF-fed rats.

**Figure 3:**
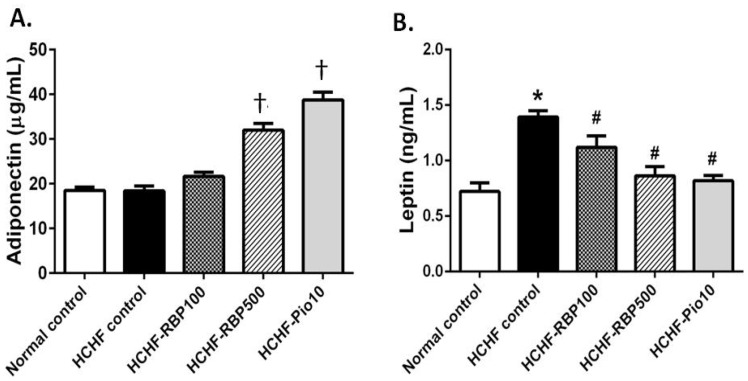
Effects of RBP on serum adeponectin (**A**) and leptin (**B**) in HCHF-fed rats. Oral administration of RBP 500 mg/kg or pioglitazone 10 mg/kg daily for 6 weeks significantly increased serum adiponectin relative to normal control, and decreased serum leptin relative to the HCHF control group. (^†^: *p* < 0.05, significant increase as compared to HCHF-control group; *: *p* < 0.05, significant increase as compared to normal controls; ^#^: *p* < 0.05, significant decrease as compared to HCHF-control group).

The serum adiponectin in the HCHF-control group was comparable to that of the normal control group. However, administrations of RBP 500 mg/kg and pioglitazone significantly increased the level of serum adiponectin (Figure 3A). The HCHF-fed rats had a significantly elevated level of leptin (Figure 3B), whereas administration of RBP (100 and 500 mg/kg) or pioglitazone restored the level of leptin in HCHF-fed rats to that of the normal control group.

### 3.5. Effect of RBP on Adipocytes Ppar-*γ* Expression

**Figure 4:**
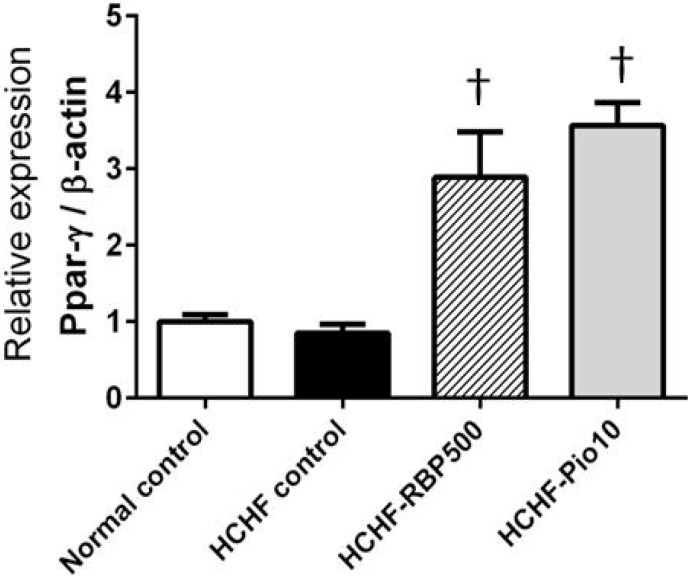
Effects of RBP on expression of adipocyte *Ppar-*γ gene in HCHF-fed rats. Oral administration of RBP 500 mg/kg or pioglitazone 10 mg/kg daily for 6 weeks significantly increased *Ppar-*γ expression (^†^: *p* < 0.05, significant increase as compared to the HCHF-control group).

PPAR-γ functions to trigger the secretion of adiponectin from adipose tissues. Adiponectin increases fatty acid oxidation in liver and skeletal muscle, and improves insulin sensitivity. We found that expression of *Ppar-*γ in intra-abdominal fat cells in the HCHF-control group was slightly decreased, but interestingly, was significantly increased in RBP 500 mg/kg or pioglitazone treated groups (Figure 4). These results reveal that RBP may attenuate insulin resistance by enhancing the expression of *Ppar-*γ*.*

### 3.6. Effect of RBP on Expression of Liver Lipogenic Genes; Srebf1 and Fasn

**Figure 5:**
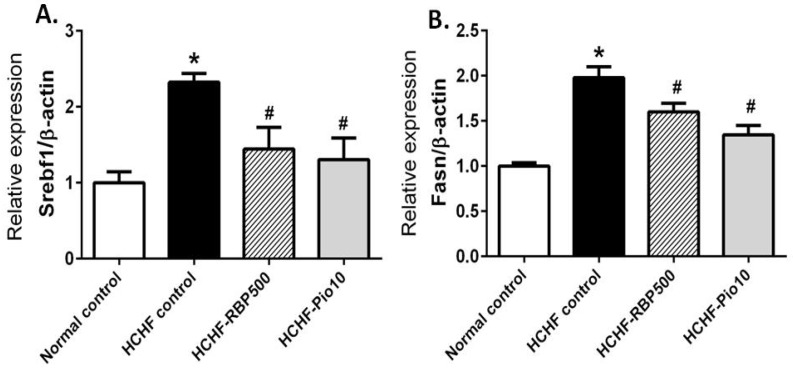
Effects of RBP on expression of lipogenic genes *Srebf1* (**A**) and *Fasn* (**B**) in HCHF-fed rats. Oral administration of RBP 500 mg/kg or pioglitazone 10 mg/kg daily for 6 weeks significantly decreased the expression of *Srebf1* and *Fasn*. (*: *p* < 0.05, significant increase as compared to normal controls; ^#^: *p* < 0.05, significant decrease as compared to HCHF-control group).

To gain insight into the effect of RBP on lipid metabolism, we investigated the alteration in expression of the transcriptional factor involved in *de novo* lipogenesis in the liver, *i.e.*, *Srebf1* and its downstream gene, the fatty acid synthase gene *(Fasn)*, by RT-qPCR. Consistent with high-fat feeding, the expression of *Srebf1* was significantly greater in the HCHF-fed rats than in the normal control group. Interestingly, the expression of *Srebf1* was significantly decreased in HCHF-fed rats receiving RBP and pioglitazone (Figure 5A). Expression of the downstream gene *Fasn* was also significantly increased in HCHF-fed rats and partially reversed by RBP 500 mg/kg or pioglitazone treatments (Figure 5B).

### 3.7. Effect of RBP on Expression of Inflammatory Genes; Il-6, Tnf-*α*, Mcp-1, Nos2 and Anti-inflammatory Gene Il-10

**Figure 6:**
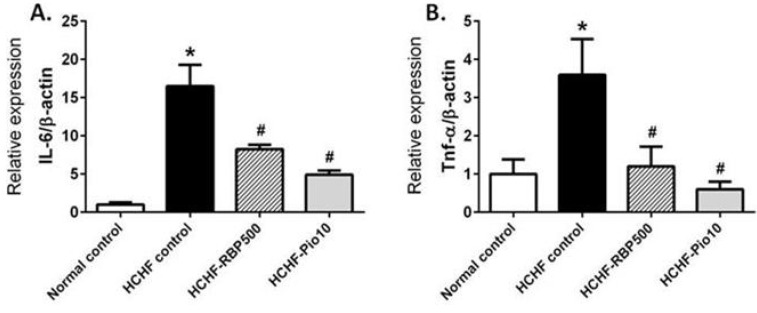

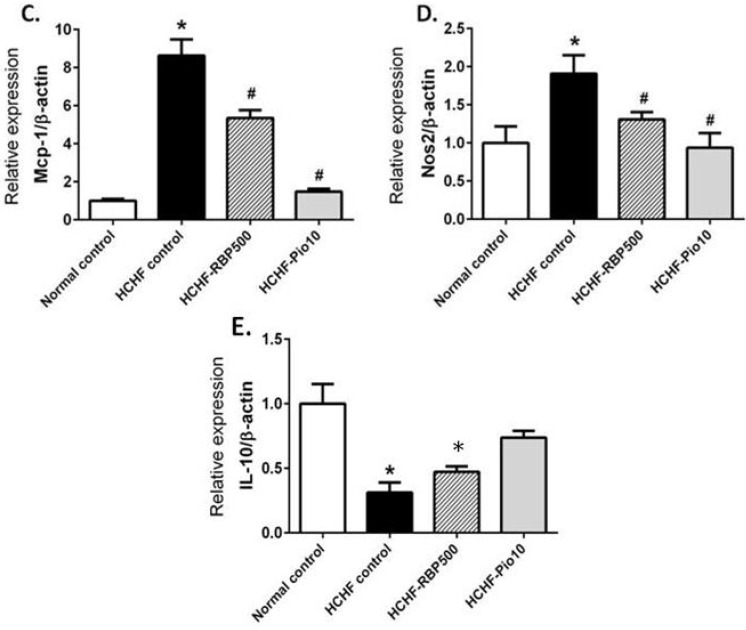
Effects of RBP on expression levels of inflammatory genes *Il-6* (**A**); *Tnf-*α (**B**); *Mcp-1* (**C**); *Nos2* (**D**) and *Il-10* (**E**) in HCHF-fed rats. Oral administration of RBP 500 mg/kg or pioglitazone 10 mg/kg daily for 6 weeks significantly decreased the expression of *Il-6*, *Tnf-*α, *Mcp-1* and *Nos2*. RBP 500 tended to increase expression of the anti-inflammatory gene *Il-10* as compared to the HCHF-control group (*: *p* < 0.05, significant increase as compared to normal controls; ^#^: *p* < 0.05, significant decrease as compared to HCHF-control group).

Low-grade chronic inflammation is related to insulin resistance in metabolic syndrome. Thus, we investigated the effects of RBP on the expression levels of proinflammatory and anti-inflammatory genes in intra-abdominal fat cells. Adipose tissues from the HCHF-control group showed a significant increase in the mRNA levels of *Il-6*, *Tnf-*α, *Mcp-1* and *Nos2* (Figure 6). RBP and pioglitazone significantly decreased the expression of all proinflammatory genes (Figure 6A–D). On the other hand, the expression of the anti-inflammatory gene *Il-10* was significant decreased in the HCHF-control group. Administration of RBP or pioglitazone produced an increase in the expression of *Il-10* (Figure 6E). Together, these results suggest that RBP may improve insulin resistance via, at least in part, decreasing inflammatory cytokine induced-insulin resistance in HCHF-fed rats.

## 4. Discussion

The present study demonstrated that chronic consumption of a HCHF diet can cause elevated fasting blood glucose, impaired glucose tolerance, dyslipidemia and insulin resistance, which are all hallmarks of metabolic syndrome. These changes are also risk factors for cardiovascular diseases and Type 2 diabetes mellitus [33]. We found that ingestion of RBP can ameliorate these risk factors in HCHF-diet induced metabolic syndrome in rats.

The increased blood glucose together with increased serum insulin level clearly suggest that insulin action on glucose regulation was impaired in HCHF-fed rats. The degree of insulin resistance was quite high as indicated by a high HOMA-IR scores. The average body weight of HCHF-fed rats was lower than that of the normal diet fed rats, which was discrepant from the other report [22]. This may be due to a relatively low food intake as compared to the normal diet control group, and the fact that the animals ate quite a low amount of HCHF pellets is probably due to the food’s texture with high fat making it less palatable to rats. However, the relative weights of liver and retroperitoneal fat, and other serum parameters including fasting blood glucose, HOMA-IR and lipid profile, are consistent with metabolic syndrome.

The treatment of HCHF-fed rats with RBP resulted in decreased blood glucose, serum insulin and HOMA-IR scores. This indicated that RBP improves insulin resistance in animals with metabolic syndrome. Dyslipidemia (high TC, LDL and TG) developed in HCHF-fed rats. RBP treatment caused a decrease in all of these as well as a decrease in fat mass. These effects may be related to the improved insulin action in peripheral tissue, *i.e.*, liver, white adipose tissue and skeletal muscle. One pathway of *de novo* lipogenesis is regulated by transcription factor SREBP-1C and its downstream gene, *Fasn*. Upregulation of these is usually found in any stress condition such as excessive intake of glucose, carbohydrates or fat. These conditions contribute to FFA induced-insulin resistance [23]. Thus we investigated the role of RBP on the expression levels of *Srebf1* and *Fasn* in liver tissues. We found that treatment with RBP can suppress HCHF diet-induced elevated expression of *Srebf1* and *Fasn* with consequent alleviation of dyslipidemia. Furthermore, the improvement in lipid metabolism by RBP may play an important role in attenuation of insulin resistance.

Chronic consumption of HCHF diet causes a stress condition in association with the abnormal adipokine secretion, elevation of serum leptin and reduction of serum adiponectin. Leptin is involved in the control of insulin-sensitivity via inhibition of lipogenesis and gluconeogenesis [15]. However, after prolonged exposure to a high-fat diet, the animals become resistant to leptin, as shown by a high leptin level and lack of response to additional exogenous leptin [34]. In this study the increased serum leptin level may be a response to the high-energy diet and also to the leptin resistance situation (high levels of leptin cannot produce a significant effect). Interestingly, this was ameliorated by RBP treatment. Adiponectin is well known as an anti-diabetic and insulin-sensitizing adipokine [35]. It causes increases in glucose uptake and fatty acid oxidation, and a decrease in hepatic gluconeogenesis. In our experiment, the level of adiponectin of the HCHF-fed control animals was not altered, which might be due to the fact that the secretion function of adipocytes was not extensively impaired. However, the treatment of HCHF-fed rats with RBP produced an elevation of adiponectin levels and this effect of RBP may result in improving insulin sensitivity and lowering of blood glucose.

In obese humans and HCHF-fed rodent models, the expression of proinflammatory adipokines is enhanced, and these are belived to induce insulin resistance [36,37]. In addition to fat cells, it has been shown that in obesity white adipose tissue is infiltrated by macrophages, which may also be a major source of proinflammatory cytokines, TNF-α, IL-6, MCP-1 and iNOS. These can cause the downregulation of the insulin signaling pathway in skeletal muscle and liver cells [36]. Consistent with previous studies, we found over-expression of proinflammatory genes *Tnf-*α, *Il-6* and *Nos2* from WAT of HCHF-fed rats. Moreover, expression of *Mcp-1* displayed a considerable increase concomitant with a very significant increase in the serum insulin level at week 18 in the HCHF-control group (MCP-1 is a recruiting factor for circulating monocytes). These findings support the causative role of chemokines in the observed insulin resistance. The treatment with RBP caused a decreased expression level of the proinflammatory genes *Tnf-*α, *Il-6*, *Nos2* and *Mcp-1* and, interestingly, tended to promote the expression of the anti-inflammatory gene *Il-10*. Consistent with our study is the demonstration of an insulin-sensitizing effect of RBP in inflammatory cytokine-induced IR in HepG2 [24].

Another important glucose and fat metabolism regulating molecule is PPAR-γ. This is the target of anti-diabetic drugs such as pioglitazone. An elevation of PPAR-γ improves insulin sensitivity and glucose metabolism, and reduces inflammation [38,39]. Our results showed that the HCHF-diet tended to decrease the expression of *Ppar-*γ in adipocytes whereas treatment with RBP and pioglitazone promoted the up-regulation of *Ppar-*γ in fat cells. Thus it is plausible that RBP caused an increase in insulin sensitivity via upregulation of *Ppar-*γ. In addition, the upregulation of *Ppar-*γ may subsequently suppress expression of the proinflammatory cytokine genes as we also found decreased expression of *Tnf-*α, *Il-6* and *Nos2* in animals treated with RBP or pioglitazone. Numerous studies have suggested that the PPAR-γ activation promotes the secretion of beneficial adipokines such as adiponectin [40,41,42]. Since RBP could cause the up-regulation of *Ppar-*γ expression, this may imply that RBP caused an increase in adiponectin secretion via upregulation of *Ppar-*γ.

Overall, this study suggests that RBP treatment improves glucose and fat metabolism in HCHF diet-induced metabolic syndrome. Even though protein is the major component of RBP, it could not rule out the possibility that some substances in the extract other than peptide may contribute in some minor way to the overall effects of RBP. In conclusion, RBP may lead to amelioration of insulin resistance through modulation of adipokine secretions, upregulation of the *Ppar-*γ gene, and downregulation of lipogenesis and proinflammatory cytokine genes. Therefore, RBP may have therapeutic effects against high-carbohydrate or high-fat diet-associated insulin resistance and metabolic syndrome, and it is highly plausible that RBP can be developed as a functional food for metabolic syndrome patients.

## Abbreviations

AUC: Area under the curve
E/S: Enzyme per substrate
Fasn: Fatty acid synthase
FBG: Fasting blood glucose
FFA: Free fatty acid
HCHF: High carbohydrate-High fat diet
HOMA-IR: Homeostatic model assessment of insulin resistance
IL-6: Interleukin 6
IL-10: Interleukin 10
IR: Insulin resistance
IRS-1: Insulin receptor substrate-1
iNos: Inducible nitric oxide synthase
Mcp-1: Monocyte chemoattractant protein-1
MS: Metabolic syndrome
mRNA: messenger RNA
Nos2: Nitric oxide synthase2
OGTT: Oral glucose tolerance test
PPAR-γ: Peroxisome proliferator-activated receptor-gamma
RT-qPCR: Real-time quantitative reverse transcription polymerase chain reaction
RBP: Rice bran protein hydrolysates
SOCS3: Suppressor of cytokine signaling 3
Srebp-1c: Sterol regulatory element-binding proteins-1c
STAT3: Signal transducer and activator of transcription 3
T2DM: Type 2 Diabetes mellitus
Tnf-α: Tumor necrosis factor- alpha
WAT: White adipose tissue
